# Why is the H_3_^+^ hot spot above Jupiter's Great Red Spot so hot?

**DOI:** 10.1098/rsta.2018.0407

**Published:** 2019-08-05

**Authors:** L. C. Ray, C. T. S. Lorch, J. O'Donoghue, J. N. Yates, S. V. Badman, C. G. A. Smith, T. S. Stallard

**Affiliations:** 1 Space & Planetary Physics, Lancaster University, Lancaster, UK; 2 Goddard Space Flight Center, NASA, Greenbelt, MD, USA; 3 European Space Agency, ESAC, Villanueva de la Canada, Spain; 4 Physics Department, The Brooksbank School, Elland, UK; 5 Department of Physics and Astronomy, University of Leicester, Leicester, UK

**Keywords:** H_3_^+^, Jupiter, atmospheric electrodynamics, Great Red Spot

## Abstract

Recent observations of Jupiter's Great Red Spot indicate that the thermosphere above the storm is hotter than its surroundings by more than 700 K. Possible suggested sources for this heating have thus far included atmospheric gravity waves and lightning-driven acoustic waves. Here, we propose that Joule heating, driven by Great Red Spot vorticity penetrating up into the lower stratosphere and coupling to the thermosphere, may contribute to the large observed temperatures. The strength of Joule heating will depend on the local inclination angle of the magnetic field and thus the observed emissions and inferred temperatures should vary with planetary longitude as the Great Red Spot tracks across the planet.

This article is part of a discussion meeting issue ‘Advances in hydrogen molecular ions: H_3_^+^, H_5_^+^ and beyond’.

## 1. Introduction

Jupiter's Great Red Spot (GRS) is thought to be the longest-lived storm in the Solar System. Centred at approximately 20° south latitude, the GRS is an anticyclonic feature with an approximate size of 22 000 km × 11 000 km in longitude and latitude, respectively [1]. The storm sits in a retrograde, westward jet, which is diverted to the north. The southern boundary is a prograde, eastward jet.

The majority of the GRS vorticity is contained in a ring approximately 75–80% of the storm's radius [1,2]. The velocity shears relative to the average background zonal flows maximize at 95 m s^−1^ in the northern portion of the storm ring [3]. A warm core of zero velocity populates the centre of the storm with evidence of weak cyclonic rotation in the inner region [4]. The vertical wind structure can be inferred from observations of the GRS thermal profile. Temperature gradients indicate that the winds decay with increasing altitude into the lower stratosphere [5]. The altitude of the peak velocity is not constant throughout the GRS but rather varies with latitude, increasing at southern latitudes [4–6].

**Figure 1.**
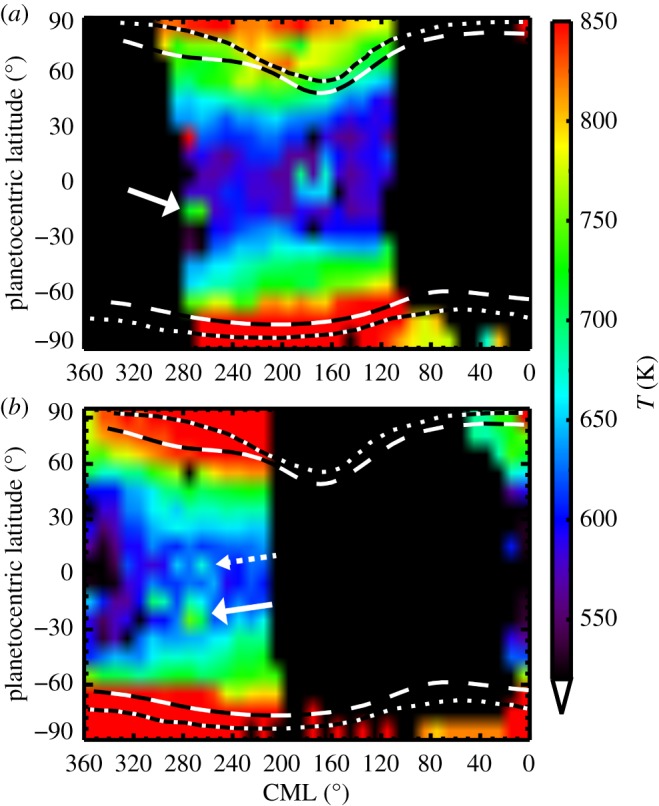
Atmospheric temperatures determined from two H_3_^+^ Keck/NIRSPEC observations from (*a*) 14 April 2016 and (*b*) 17 April 2016. The GRS is centred at 270° System III longitude. The solid white arrows denote the location of the GRS. In the 17 April 2016 image (*b*), a temperature enhancement can also be seen at the location magnetically conjugate with the GRS (dashed white arrow).

While the visible signatures of the GRS have been extensively studied over the years, the thermosphere above the storm has only recently been explored. An analysis of H_3_^+^ observations of the GRS from 2012 using the SpeX spectrometer on the NASA Infrared Telescope Facility [7] showed that the thermosphere above the storm is heated to temperatures of 1600 K. The enhanced thermospheric temperatures are concentrated above the GRS, with sharp gradients at the edges of the storm. The localized high temperatures are also evident in thermospheric temperature maps inferred from observations taken in 2016 with the Near Infrared Spectrometer on the Keck Telescope (Keck/NIRSPEC; figure 1). Interestingly, H_3_^+^ temperatures determined from the 2016 observations maximize at approximately 750 K, a reduction of approximately 50% from the 2012 observations, but are still significantly warmer than the surrounding atmosphere. Spatially, the centre of the GRS shifted in System III longitude from 246° to 270° between 2012 and 2016. Other hot spots are present in figure 1; however, we focus on the GRS in this analysis as it is a repeatedly observed feature confirmed by measurements using two different telescope facilities. The temporal variability of the mid-to-low latitude thermosphere away from the GRS may contain further clues about thermospheric heating and should be considered in future studies.

The large observed H_3_^+^ temperatures above the GRS are distinct within Jupiter's mid-to-low latitudes and go beyond the giant planet ‘energy crisis’ [8,9]. In short, the thermospheric temperatures of the giant planets are hundreds of Kelvin hotter than the approximately 150–200 K that can be produced by absorption from solar extreme ultraviolet (EUV) radiation alone [8]. At sub-auroral latitudes, temperatures inferred from H_3_^+^ observations range from 700 to 850 K [10]. At near-equatorial latitudes, *in situ* temperatures measured by the Galileo probe maximize at approximately 900 K [11]. In the auroral regions, the thermosphere reaches temperatures of 1000–1400 K [12,13].

In the auroral regions, magnetosphere–ionosphere–thermosphere coupling processes can generate heating through a plethora of mechanisms. Energetic precipitating electrons chemically heat the atmosphere through ionization and excitation [14,15]. This heat is transferred to the surrounding neutral atmosphere through neutral–neutral and ion–neutral collisions. An additional heating source is Joule heating, which arises from currents and electric fields ultimately driven by magnetospheric dynamics [16–18]. An outstanding problem though is how this energy is transported from auroral regions to low latitudes. Numerical models thus far have shown that a combination of ion drag and strong centrifugal forces confine auroral energy to the poles [19]. A recent analysis suggests that enhanced Rayleigh drag may counteract this confinement and enhance equatorward transport [20]. However, the GRS is at low magnetic latitudes and is a localized enhancement, thus we must look to other mechanisms.

Potential atmospheric sources of heating include upward-propagating gravitational waves from the lower thermosphere and acoustic waves. As gravity waves dissipate, they deposit energy into the local atmosphere. However, there is also a cooling effect at altitudes above the peak wave amplitudes. The net heating on a column of air may only be approximately 200 K, which is not enough alone to explain the observed GRS or general mid-to-low latitude temperature enhancement [21].

Acoustic waves can also propagate upwards and, through viscous dissipation, heat the atmosphere. Generated above thunderstorms, the GRS could be an ideal weather system to generate these waves. Models of acoustic waves at Jupiter suggest that they can heat the local atmosphere by tens to hundreds of Kelvin per day [22]. However, if the source of the acoustic waves is spatially limited, then the heating will be reduced owing to geometric spreading of the wave. Unfortunately, there are no direct observations of upward propagating acoustic waves at the outer planets.

Another source of heating is electrodynamic coupling between Jupiter's thermosphere and stratosphere [23]. Stratospheric winds generate electric fields in the embedded ionosphere. The associated Hall and Pedersen currents, if divergent or convergent, couple to the thermosphere along the planetary magnetic field. Joule heating dissipates energy into the upper atmosphere. Thus there is a net transfer of kinetic energy from the stratosphere to thermal energy in the thermosphere. This mechanism requires that (i) the ionosphere extends into the stratosphere and (ii) the wind flows drive divergent/convergent currents, thus requiring current closure along the magnetic field and into the thermosphere. It is this mechanism that we consider in this study.

## 2. Electrodynamic coupling and the feasibility of electric fields

The GRS is primarily a tropospheric storm, yet it extends vertically into the lower stratosphere. Thermal gradients result in the winds diminishing; however, infrared observations suggest that they persist into the lower stratosphere [5]. Galileo radio occultations of Jupiter's mid-latitude ionosphere show an ionospheric peak in the electron density at altitudes at approximately 600 km above the 1 bar level [24] near the lower boundary of the measurement. Voyager occultations of the mid-to-high latitude ionosphere show peaks in the electron density deeper in the atmosphere at altitudes from 300 to 500 km above the 1 bar level [25]. The sparsity of occultation data precludes drawing any global conclusions as to the depth of the ionosphere and the viewing geometry restricts our description to the dawn and dusk limbs. If we assume that the stratosphere–thermosphere boundary is at an altitude of approximately 360 km above the 1 bar level, or equivalently approximately 0.34 μbar [11], then we can speculate that the ionosphere penetrates into the stratosphere to explore electrodynamic heating.

In the thermosphere, the dominant ions are H^+^ and H_3_^+^. However, hydrocarbons become more important at lower altitudes. At Saturn, these hydrocarbon ions contribute strongly to the ionospheric conductivity [26] and may provide a low-altitude source of conductivity. Similar physics may apply at Jupiter. Additionally, electron conductivity, typically neglected in the thermosphere because of the low electron-neutral collision frequency, may play a larger role at lower altitudes. The electron-neutral collision frequency increases with the density of the neutral atmosphere, with the electron Pedersen conductivity maximizing where the electron gyrofrequency approaches the local electron-neutral collision frequency.

The strong wind shears generated by the GRS drive currents and electric fields in the ionosphere. The current density, **j**, can be related to the electric fields as follows:

2.1 $$j_{⊥}=\sigma_{P}(E_{⊥}+v\times B)+\sigma_{H}\hat{b}\times (E_{⊥}+v\times B)$$

and

2.2 $$j_{||}=\sigma_{||}E_{||},$$

where **E**_⊥_ is the electric field perpendicular to the magnetic field, *E*_||_ is the electric field component parallel to the magnetic field, **v** is the ionospheric bulk flow in the rest frame of the neutral atmosphere, **B** is the planetary magnetic field, and *σ*_*P*_, *σ*_*H*_, and *σ*_||_ are the local Pedersen, Hall and parallel conductivities, respectively.

Ion drag extracts kinetic energy from the local neutral winds through collisions. This can be expressed in terms of the local conductivities and electric fields as:

2.3 $$q_{\mathrm{ID}}=v\cdot (J\times B)=-\sigma_{P}(E_{⊥}+v\times B)\cdot v\times B+\sigma_{H}(vB)\cdot E_{⊥}.$$

Thermal energy is input into the atmosphere through Joule heating, *q*_JH_:

2.4 $$q_{\mathrm{JH}}=j\cdot (E+v\times B)=\sigma_{||}E_{||}^{2}+\sigma_{P}(E_{⊥}+v\times B)\cdot (E_{⊥}+v\times B).$$

Ion drag and Joule heating influence the energy balance in both the stratosphere and the thermosphere. The relative contribution of each process depends on the full conductivity tensor and the mechanisms driving the local electric fields. Any electric fields and currents generated by GRS winds are at sufficiently low magnetic latitudes that they should close within the local atmosphere or in the magnetically conjugate location of the northern hemisphere. This means that magnetospheric coupling can be ignored. In a steady-state system, in the absence of other sources of heating or cooling, energy conservation dictates that the Joule heating and ion drag terms must sum to 0 across all coupled regions.

To consider the feasibility of the proposed heating mechanism, it is necessary to understand the electric fields generated by the GRS vorticity in the low-altitude ionosphere. In this study, we focus primarily on the electric field structure to determine whether electrodynamic coupling merits further investigation as a possible thermospheric heat source above the GRS and what potential observations could provide additional evidence.

## 3. Vortex-related electric fields

**Figure 2.**
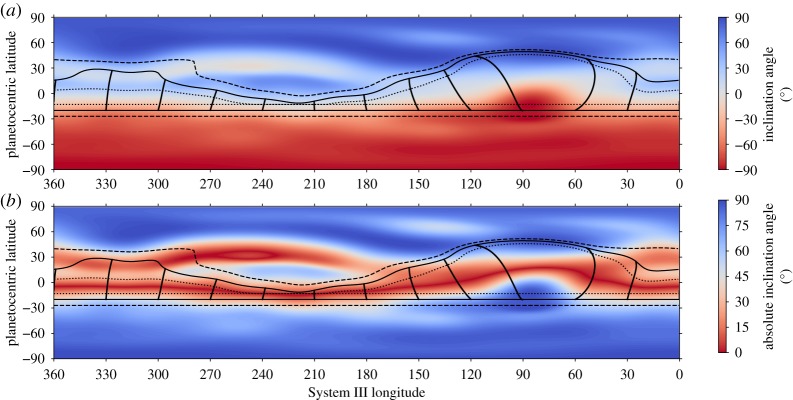
Magnetic field inclination angle (*a*) and the absolute value of the magnetic field inclination angle (*b*) at the dynamically flatted surface, 1*R*_*J*_ × (1 − cos^2^*θ*/15.4). The solid lines at − 20° track the centre of the GRS. The solid lines in the northern hemisphere show the magnetically conjugate location of the GRS footprint. The semi-vertical lines are the projection of the magnetic field from the GRS footprint to the magnetically conjugate location at intervals of 30° *λ*_*III*_. These lines highlight the longitudinal drift of the footprint. The dashed and dotted lines track −27° and −13° and their magnetically conjugate locations, respectively.

Figure 2 displays the dip angle and the absolute value of the dip angle of the planetary magnetic field from the recent JRM09 field model [27] on a 1° × 1° grid. Jupiter is dynamically flattened; hence, we consider the dip angle at a distance of 1*R*_*J*_ × (1 − cos^2^*θ*/15.4), where 1*R*_*J*_ = 7.14 × 10^7^ m and *θ* is colatitude. The solid line at −20° tracks the motion of the GRS in System III longitude as it drifts around the planet. The corresponding line in the northern hemisphere denotes the magnetically conjugate location of the GRS footprint, with the semi-vertical lines showing the trace of the magnetic field between the two footprints every 30° *λ*_*III*_ longitude. The dashed and dotted lines track the southern and northern boundaries of the GRS at −27° and −13°, with their associated conjugate locations in the northern hemisphere.

It is immediately clear from figure 2 that the GRS passes through a variety of magnetic field configurations, from a horizontal field configuration on the magnetic equator at approximately 230° *λ*_*III*_ to a near-vertical field when it passes through one of Jupiter's many magnetic anomalies near 90° *λ*_*III*_. The magnetic field magnitude at GRS latitudes ranges from approximately 4 to 6 Gauss [27, fig. 2].

**Figure 3.**
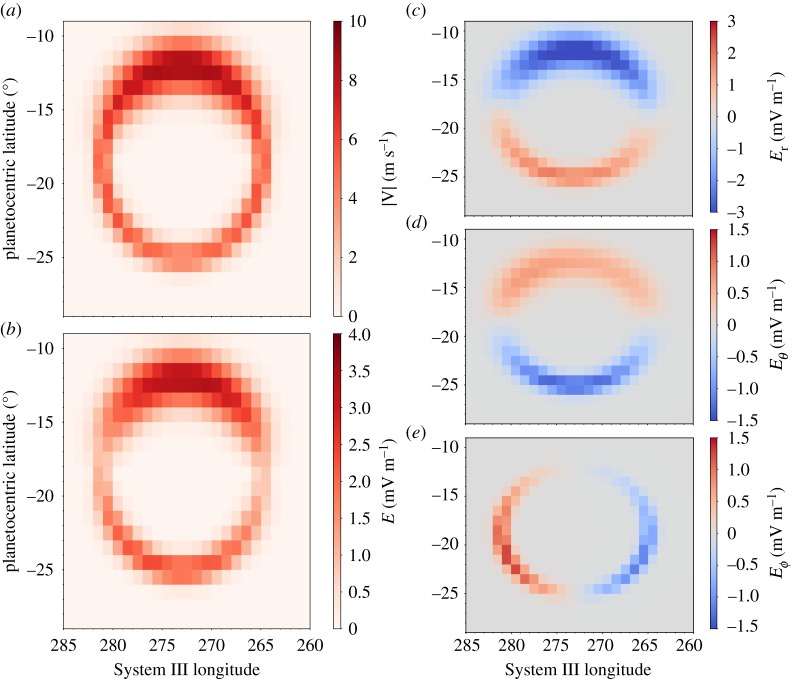
Imposed anticyclonic velocity field (*a*), electric field magnitude (*b*), and radial (*c*), meridional (*d*) and azimuthal (*e*) components of the associated **v** × **B** electric field for a GRS vortex centred at 274° *λ*_*III*_.

Owing to the decay in GRS flows with altitude [5], the wind field in the lower stratosphere is assumed to be approximately 10% of the tropospheric flows, i.e. all wind vector components are scaled to be approximately 10% of the tropospheric magnitudes. The zonal averages have been subtracted, meaning that only deviations from the ambient neutral flow remain [3]. Figure 3 shows the velocity field, electric field magnitude and electric field components for a GRS centred at 274° *λ*_*III*_. The magnitude of the stratospheric electric field, **E**, is given by **E** = **v** × **B**, where **v** is assumed to be in the rest frame of the neutral stratosphere and entirely in the horizontal plane, i.e. only azimuthal and meridional components to the velocity, and **B** is the planetary magnetic field vector [27].

The electric field magnitude maximizes on the northern edge of the GRS where the shear flows are strongest, with a minimum on the flanks. Since the velocity on the flanks is comparable to that on the southern boundary of the GRS, this minimum can be attributed to the reduction in the flow perpendicular to the magnetic field, i.e. the northward and southward flows have a larger component parallel to the magnetic field. Each electric field component exhibits a dichotomy driven by opposing flows. The radial and meridional electric fields reflect anticorotational flows along the equatorward edge of the GRS and corotational flows on the southern edge. Northward and southward flows on the flanks of the GRS generate anticorotational and corotational azimuthal electric fields, respectively.

The electric field in figure 3 is that generated in the stratosphere. In the absence of significant horizontal gradients in magnetic field-aligned electric fields, the magnetic field can be treated as an equipotential surface, i.e. perpendicular electric fields simply scale using conservation of magnetic flux. Since the thermosphere and stratosphere cover a region that is only a few hundreds to thousands of kilometres deep, we approximate that the perpendicular electric fields are constant between the two regions to first order. This assumption requires that the field-aligned conductivity dominates over the perpendicular conductivities and should be tested in future work.

### (a) Variations in GRS System III Longitude

**Figure 4.**
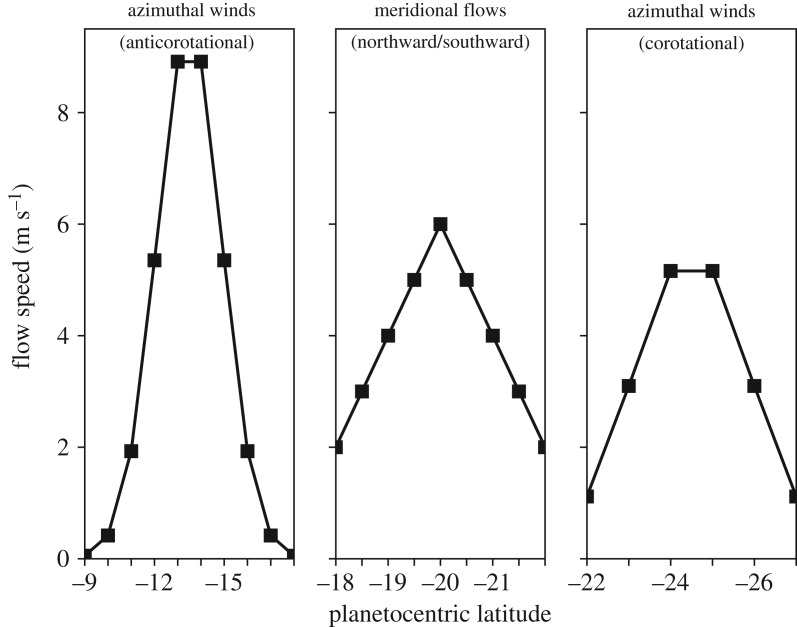
Assumed GRS winds in the stratosphere based on 10% of the flow speeds [3]. The anticorotational azimuthal winds are over a latitudinal range of −9° to −18°, the meridional flows span a latitudinal range of −18° to −22° and the corotational azimuthal winds flow between −22° and −27° latitude.

To investigate the variation in the electric field structure as the GRS drifts in System III longitude, we impose a simpler flow pattern (figure 4) where only purely azimuthal and meridional flows are considered. From −9° to −18° latitude, the imposed flow is anticorotational in the azimuthal direction. Between −18° and −22°, northward and southward meridional flows are imposed in turn to investigate both edges of the anticyclonic vortex. Finally, at the southern boundary, from −22° to −27°, there are corotational azimuthal winds. The maximum speed of the winds within each latitude band declines with increasing latitude, with the eastward winds maximizing at 9.0 m s^−1^, the meridional flows capping at 6 m s^−1^ and the westward winds peaking at 5 m s^−1^.

**Figure 5.**
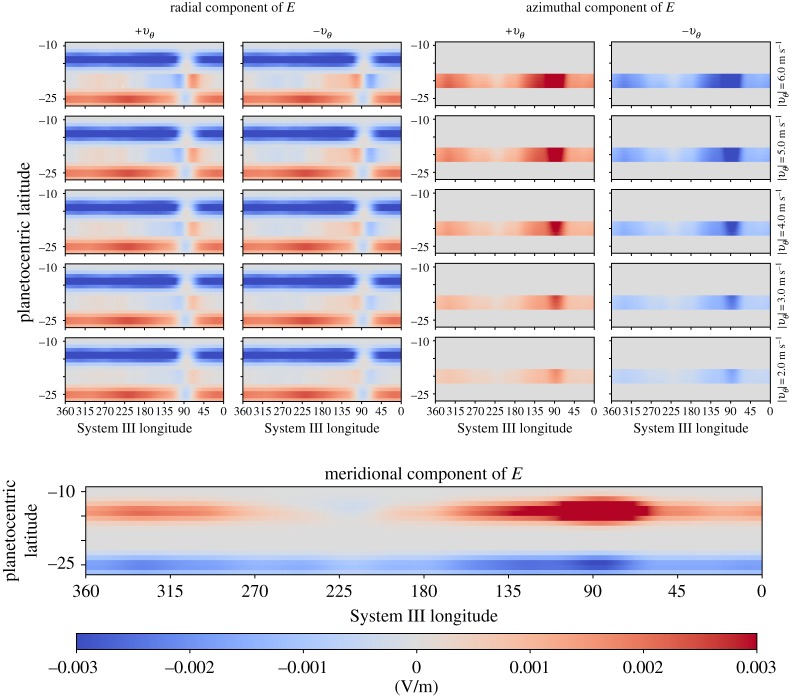
Longitudinal variation in the radial, azimuthal and meridional **v** × **B** electric field components generated by the flow patterns from figure 4. Southward (+*v*_*θ*_) and northward (−*v*_*θ*_) meridional flows are considered individually in the left and right columns, respectively, of the radial and azimuthal electric field sections.

Figure 5 shows the radial, azimuthal and meridional electric fields generated by the imposed ionospheric flows in the stratosphere. The electric field signatures of the northward and southward flows, denoted by ‘−*v*_*θ*_’ and ‘+*v*_*θ*_’, respectively, are considered at all longitudes rather than imposing successive single vortexes with longitude. This approach allows a better investigation of the longitudinal variations present in the system; however, the true electric field signature of the vortex naturally has contributions from both northward and southward flow components similar to those in figure 3. Additionally, the sharp discontinuities at the boundaries of the latitude bands presented in figure 4 do not exist in the physical system. Rather, the direction of the electric fields should smoothly vary between the azimuthal and meridional winds as evidenced in the single vortex case presented in figure 3.

From figure 5, it is obvious that the magnetic field geometry drives the strong System III variations in the electric field direction and magnitude. Away from the anomaly region around 90° *λ*_*III*_, the radial component of the electric field dominates, pointing inwards on the northern vortex edge and outwards along the southern edge. A smaller radial component is driven by the meridional flows; the azimuthal component of the electric field dominates in the middle region of the vortex. The azimuthal winds also result in a meridional component to the electric field. Near the magnetic equator, approximately 225° *λ*_*III*_, a structure appears where oppositely directed meridional electric field components converge, which could result in currents diverging from the northern edge of the vortex along the magnetic field.

Between −18° and −22° latitude, the azimuthal electric fields at the eastward and westward flanks of the GRS are oppositely directed because of the northward and southward winds. Interestingly, near 90° *λ*_*III*_, the magnetic field structure is such that the northward and southward winds drive radial electric fields of similar orientation. However, there would be a region of negligible electric field in the centre of the vortex where there is little vorticity. This discontinuity could again result in currents converging/diverging along the magnetic field into/out of the vortex.

## 4. Implications for thermospheric heating

The electric fields generated by the assumed GRS vortex winds in the lower stratosphere are complex and strongly vary with planetary longitude. For a single vortex, e.g. a GRS centred at 274° as in figure 3, the electric field is concentrated in a ring spanning approximately 20° longitude. The magnitudes of the electric fields generated by the shears shown in figure 4 are of the order of mV m^−1^. Sharp gradients in the electric field at the edges of the GRS as well as those near the central non-rotating core region will drive field-aligned currents. Since the GRS is a long-lived storm system that drifts slowly with respect to System III longitude, temporal changes in the local magnetic field configuration are likely to be negligible compared with these strong spatial gradients.

While the electric fields are a critical component to electrodynamic coupling, we must also consider the nature of the currents in the stratosphere. A curl in the vorticity will drive Pedersen currents that must close along electric fields, while a divergence in the flow generates Hall currents. Thus, the non-uniform velocity around GRS leads to Hall currents, while gradients perpendicular to the flow produce Pedersen currents. In addition to the horizontal flows here, observations of the GRS indicate that it is tilted, with the northern edge shallower than the southern [5]. This additional velocity component in the radial direction would further complicate the currents and electric field patterns in the local atmosphere.

Substantial Hall and Pedersen conductivities are required for GRS flows to electrodynamically heat the thermosphere. The Hall conductivity will extract the kinetic energy from the storm winds, while the Pedersen conductivity, along with any parallel conductivity in the system, controls the resistive heating of the upper atmosphere. Therefore, to optimize thermospheric heating and energy extraction from the stratosphere, the peak in the Hall conductivity should occur at a lower altitude than the Pedersen conductivity.

There are currently few models of Jupiter's mid-to-low latitude ionosphere and the conductivity is not well understood. Typical values for the height-integrated conductivity generated by solar illumination at high latitudes are approximately 0.0006 mho [28], orders of magnitude lower than that generated indirectly by auroral electron precipitation. At mid-to-low latitudes, the conductivity generated by solar EUV flux may be higher as a result of the angle of incidence of incoming radiation. At low altitudes of pressures between 2 × 10^−6^ and 2 × 10^−7^ bars, atmospheric models of the equatorial dayside conductivity profile show two peaks of 1 × 10^−8^ mho m^−1^ and 1 × 10^−7^ mho m^−1^ [15]. However, these values do not include any contributions from electron-neutral collisions, which could be important at stratospheric altitudes.

Assuming a simplified atmospheric structure consisting of a 100 km thick slab stratosphere and a 300 km thick slab thermosphere, with Pedersen conductivities of 1 × 10^−8^ mho m^−1^ and 1 × 10^−7^ mho m^−1^, respectively, we can estimate the power deposited into the thermosphere by the GRS flows. Ignoring both externally imposed electric fields and magnetic field-aligned electric fields, the power deposited in the thermosphere for a vortex centred at ∼274° *λ*_*III*_ longitude is approximately 0.1 μW m^−2^. This is three orders of magnitude smaller than auroral energy deposition near the poles. However, a more rigorous analysis is required to further quantify the heating.

The longitudinal shift in direction of the electric fields could lead to interesting effects. The radially outward-directed electric field associated with the corotational flows on the southern edge of the storm may lead to an electrostatic upwelling of H_3_^+^. This could artificially inflate the temperatures inferred from ground-based observations as the increased density of H_3_^+^ at higher altitude could skew the intensity of the observed emission. This effect would decrease near the magnetic anomaly near 90° *λ* where the vertically directed magnetic field results in largely meridional electric fields. The radial electric fields are strongest near the magnetic equator and thus this effect could explain the much larger temperatures from the 2012 observations [7] relative to the 2016 observations presented here because the GRS was nearer to the magnetic equator in 2012.

Another observational consequence of this heating mechanism is potential conjugate emission in the northern hemisphere. The conjugate traces shown in figure 2 from the longitudinal motion of the GRS indicate where additional heating might exist. If the magnitude of the parallel electric fields generated by the gradients at the edges of the vortex is small relative to the perpendicular electric fields, then we can approximate the magnetic field lines intersecting the GRS as equipotentials. Field-aligned currents resulting from the divergence of perpendicular currents can close in the opposite hemisphere, generating Joule heating. This could result in a conjugate H_3_^+^ spot. There are indications of a conjugate spot in figure 1*b*; however, the robustness of this interpretation needs to be confirmed by further observations. Unfortunately, during the 2012 observations the GRS was on the magnetic equator and therefore we would not expect to see any conjugate emission.

## 5. Conclusion

H_3_^+^ observations of Jupiter's thermosphere show enhanced heating above the GRS. This long-lived storm generates shear winds in the troposphere that extend into the stratosphere. Electrodynamic heating due to ionospheric vortexes driven by the GRS may contribute to the large observed thermospheric temperatures. These winds can generate electric fields of the order of mV m^−1^ with strong spatial variations along the vortex. Ion drag processes associated with these electric fields can extract kinetic energy from the stratosphere and deposit it in the thermosphere through resistive heating. Additionally, the complex planetary magnetic field structure leads to longitudinal variations in the electric fields that could lead to longitudinal System III variations in the heating. We have shown that this mechanism merits further investigation, which should include modelling of the mid-to-low latitude conductivity. Future H_3_^+^ observations could shed light on this heating mechanism by (i) providing longitudinal dependences of the heating and (ii) confirming the existence of conjugate emission.

The complexity of Jupiter's atmosphere–ionosphere is such that multiple heating mechanisms are likely to be present. However, even a contribution of approximately 10–100 K could assist in our understanding of Jupiter's thermosphere. H_3_^+^ observations provide crucial evidence to test and constrain our theories.

## Data accessibility

This article has no additional data.
